# Silk Fibroin Bioink for 3D Printing in Tissue Regeneration: Controlled Release of MSC extracellular Vesicles

**DOI:** 10.3390/pharmaceutics15020383

**Published:** 2023-01-22

**Authors:** Elia Bari, Giulia Maria Di Gravina, Franca Scocozza, Sara Perteghella, Benedetta Frongia, Sara Tengattini, Lorena Segale, Maria Luisa Torre, Michele Conti

**Affiliations:** 1 Department of Pharmaceutical Sciences, University of Piemonte Orientale, Largo Donegani 2/3, 28100 Novara, Italy; 2 Department of Industrial and Information Engineering, University of Pavia, Via Ferrata 5, 27100 Pavia, Italy; 3 Department of Civil Engineering and Architecture, University of Pavia, Via Ferrata 3, 27100 Pavia, Italy; 4 Department of Drug Sciences, University of Pavia, Via Taramelli 12, 27100 Pavia, Italy; 5 PharmaExceed s.r.l., Piazza Castello 19, 27100 Pavia, Italy

**Keywords:** silk fibroin, sodium alginate, controlled release, 3D bioprinting, bioink, MSC-secretome, MSC-extracellular vesicles

## Abstract

Sodium alginate (SA)-based hydrogels are often employed as bioink for three-dimensional (3D) scaffold bioprinting. They offer a suitable environment for cell proliferation and differentiation during tissue regeneration and also control the release of growth factors and mesenchymal stem cell secretome, which is useful for scaffold biointegration. However, such hydrogels show poor mechanical properties, fast-release kinetics, and low biological performance, hampering their successful clinical application. In this work, silk fibroin (SF), a protein with excellent biomechanical properties frequently used for controlled drug release, was blended with SA to obtain improved bioink and scaffold properties. Firstly, we produced a printable SA solution containing SF capable of the conformational change from Silk I (random coil) to Silk II (β-sheet): this transition is a fundamental condition to improve the scaffold’s mechanical properties. Then, the SA-SF blends’ printability and shape fidelity were demonstrated, and mechanical characterization of the printed hydrogels was performed: SF significantly increased compressive elastic modulus, while no influence on tensile response was detected. Finally, the release profile of Lyosecretome—a freeze-dried formulation of MSC-secretome containing extracellular vesicles (EV)—from scaffolds was determined: SF not only dramatically slowed the EV release rate, but also modified the kinetics and mechanism release with respect to the baseline of SA hydrogel. Overall, these results lay the foundation for the development of SA-SF bioinks with modulable mechanical and EV-release properties, and their application in 3D scaffold printing.

## 1. Introduction

Three-dimensional (3D) scaffolds for application in tissue engineering (TE) are generally employed as support to improve the proliferation and differentiation of cells seeded on the scaffold or for in vivo colonization when implanted. Among the different techniques employed for scaffold preparation, 3D printing is promising as it allows production of complex scaffolds with intricately sophisticated biomimetic 3D structures capable of promoting functional tissue regeneration [1,2,3]. Specifically, during 3D printing, thin layers of a polymer, natural or synthetic in origin, are deposited in succession to form a 3D structure [4,5,6,7,8] with a fully interconnected porous network that allows cell-to-cell interactions and efficient transport and exchange of oxygen, nutrients, and metabolites [9,10,11]. Preferably, the employed materials should be cytocompatible, mechanically compatible with the target tissue, and biodegradable with a rate that matches neotissue formation so that the scaffold fully degrades only after the regenerated tissue has been formed [12]. To further optimize biointegration in vivo, the scaffold may also contain bioactive signalling molecules, such as cytokines and growth factors, to support the formation of the desired tissue. For this purpose, mesenchymal stem cell (MSC) secretome may be used, as it is rich in growth factors, cytokines, and oligonucleotides, partially encapsulated into extracellular vesicles (EVs), that can sustain new tissue formation [13,14,15,16,17,18]. When such bioactive molecules are present, the scaffold also plays a critical role in retaining them at the implantation site for a certain time and in controlling their release, which may happen rapidly [19] or slowly [20], in different cases.

For all these purposes, hydrogels from naturally derived polymers are often chosen, such as collagen, chitosan, sodium alginate (SA), and hyaluronic acid. When dispersed in water, such polymers create thick network structures which retain high water content. Their application can (i) support encapsulation of hydrophilic molecules without denaturation and aggregation [21], (ii) hamper penetration of enzymes, thus preventing premature degradation [22], (iii) control the release rate of the encapsulated molecules as a consequence of the degradability or porosity of the network structure [23,24,25]. Moreover, hydrogels offer an environment for cells to colonize, reside, and grow, and when using polymers able to respond to external stimuli (e.g., light, heat, electricity, magnetic fields, pH), effective shape-morphing, mechanical, and biological outputs can be obtained by replying to the body’s internal environment and biological cues [26].

Despite all these significant advantages, hydrogels have shown drawbacks when used in bioprinting, including poor mechanical properties or fast-release kinetics that hamper their success in clinical applications. One strategy to overcome this may be to create scaffolds with hybrid structures, combining materials with different characteristics and properties. For example, we recently fabricated a hybrid scaffold by coprinting polycaprolactone (PCL), a thermoplastic material able to provide mechanical resistance, and SA hydrogel containing lyosecretome—a freeze-dried formulation of MSC-secretome—to obtain an improved biological response [27]. Similarly, a combination of different components in the same hydrogel may provide another method to enhance the general features of the scaffold. For example, SA has been mixed with many other materials to improve its low mechanical properties, biological response, and fast degradation [28]. Among these, silk fibroin (SF) may represent a suitable candidate due to its good mechanical properties, including high tensile strength and modulus [29], together with its excellent biocompatibility, non-immunogenicity [30,31,32,33,34], biodegradability [35], and intrinsic anti-inflammatory activity [36,37,38]. SF is a high molecular weight protein (200–300 kDa or more) in which crystalline regions (β-sheet and α-helix) are connected by amorphous or non-crystalline regions (random coil and β-turn) [39]. Depending on the type and abundance of crystalline and amorphous regions, SF has a Silk I (a mix of a random coil, α-helix domains, and β-turn structures), Silk II (abundant β-sheet structures), or Silk III (left-oriented triple helix structure) conformation [40,41,42]. Despite being widely employed as a biomaterial in tissue engineering, the literature reports limited examples of SF in bioprinting [43]. Indeed, many problems still need to be addressed, including low viscosity and, more importantly, the difficulty of 3D printing as β-sheet structures can clog the needles during the printing process [44,45].

This work aimed to obtain a bioink made of SA and SF and defined by chemical crosslinking, for 3D bioprinting applications. Initially, the preparation process of SF was optimized by adjusting the degumming time to achieve a printable protein solution. For this reason, blends of SA and SF, degummed at different times (i.e., 1, 2, and 4 h), were characterized in terms of printability and shape fidelity. Moreover, tensile and compressive mechanical tests were conducted to reveal how the SF component and the crosslinking method could influence the mechanical performance of the material. Finally, the SA-SF hydrogels were loaded with lyosecretome to investigate the release of the lipid components of the secretome (EVs) over time.

## 2. Materials and Methods

### 2.1. Materials

Deionized water was obtained from a Milli-Q^®^ Integral purification system from Merck KGaA (Darmstadt, Germany). Natriumazide and sodium sulphate were from Carlo Erba Reagenti (Milan, Italy). Potassium dihydrogen phosphate, ribonuclease A, β-lactoglobulin, bovine serum albumin, sodium alginate, sodium carbonate, lithium bromide, calcium chloride, protamine, potassium chloride, and thyroglobulin were from Sigma–Aldrich (Milan, Italy). Rituximab was purchased as MabThera from Roche (Basel, Switzerland).

### 2.2. Silk Fibroin Preparation and Characterization

#### 2.2.1. Silk Fibroin Extraction and Solubilization

To extract SF, the *Bombyx mori* cocoons were cut into pieces of 1 × 1 cm and boiled in 0.02 M sodium carbonate (Na_2_CO_3_) for 30 min [46,47], 1 h, 2 h, or 4 h (Table 1).

After being washed in distilled water (37 °C), the degummed fibers were dried at room temperature and then treated with 9.3-M LiBr aqueous solution at 60 °C for 4 h to dissolve SF. LiBr was then eliminated by dialysis against distilled water using dialysis cellulose tubes with a molecular weight cut-off (MWCO) of 3–5 kDa (Spectrum Laboratories, Milan, Italy). The dialysis was conducted at room temperature for 72 h, and the SF final concentration was determined by freeze-drying known SF volumes (Modulyo^®^ Edwards Freeze dryer, Kingston, NY, USA) at −50 °C, 8 × 10^−1^ mbar for 72 h. For the further test, SF was diluted in deionized water, reaching a final concentration of 5% *w*/*v*.

#### 2.2.2. Size Exclusion Chromatography (SEC)-UV Analysis

The molecular weights of SF degummed for different times were estimated by SEC-UV analysis. Chromatographic separations were performed on an Agilent HPLC series 1200 system (Santa Clara, CA, USA), equipped with mobile phase online degasser, quaternary pump, autosampler, thermostated column compartment, and diode array detector. For data acquisition, the ChemStation software version Rev. B.04.01 (Agilent Technologies, Santa Clara, CA, USA) was used. The analytical method, previously reported [48], entails the use of a TSKgel SuperSW3000 (4.6 × 300 mm; Tosoh Bioscience, Tokyo, Japan) and a mobile phase composed of 0.1 M Na_2_SO_4_ and 0.05% (*w*/*v*) NaN_3_ in 0.1 M phosphate buffer, pH 6.7. Flow rate, column temperature, and injection volume were set at 350 μL/min, 25 °C, and 5 μL, respectively. UV absorption was monitored at 280 nm. For MW estimation, a calibration curve was constructed using protein standards (ribonuclease A, 14 kDa; β-lactoglobulin (dimeric form), 37 kDa; bovine serum albumin, 67 kDa; rituximab, 145 kDa; thyroglobulin, 660 kDa; y = −0.9949 x + 4.9497; R^2^ = 0.9934). SF samples were diluted with water to a final concentration of 0.33% *w*/*v* before injection, and analyzed in triplicate.

#### 2.2.3. Physico-Chemical Characterization of SF

Infrared spectra were recorded using an Alpha II FT-IR spectrometer equipped with a platinum attenuated total internal reflectance (ATR) module (Bruker, Rosenheim, Germany); data were elaborated using Opus 7.8 software. Collection was performed on SF fibres (after the different degumming times) and the SF solution after exposure to KCl 20% *w*/*v* (to induce Silk I → SilK II conformation change) and being left dry.

### 2.3. Hydrogels and Crosslinking Solution Preparation

Four hydrogel formulations were prepared, as reported in Table 2, and analyzed. Three were composed of 10% *w*/*v* SA and 5% *w*/*v* SF, differing in SF degumming time. The preparation protocol involved adding and dissolving SA powder into an SF solution. The fourth composition (control) was formed of only 10% *w*/*v* SA, powdered and dissolved in distilled water. All the formulations were mixed and homogenized manually and using a rotational shaker for 10 min. Finally, considering the importance of sterile scaffolds, all bioink formulations were pasteurized at 72 °C for 1 h according to the procedure previously reported [16].

The crosslinking solution was prepared by dissolving calcium chloride (CaCl_2_) 2% *w*/*v*, protamine 5% *w*/*v*, and potassium chloride (KCl) 20% *w*/*v* powders in distilled water. The solution was stirred at 40 °C using a magnetic plate. In particular, CaCl_2_ and protamine were employed to crosslink SA, and KCl to induce the conformational change of SF from Silk I to Silk II.

### 2.4. Assessment of SA-SF Hydrogel Printability and Shape Fidelity

#### 2.4.1. 3D Bioprinter and Printing Process

Printability and shape fidelity of the SA-SF hydrogel were assessed using CELLINK INKREDIBLE+, an extrusion-based 3D bioprinter. The process involved a 3D virtual geometry translated into machine instructions using a slicing software, which generates the coordinates of the printing head in each layer along with appropriate instructions (i.e., G-code). G-code also defines the velocity of the printhead movement. The positioning system for the printing head has a 10 microns resolution in the three axes. Before starting printing, the printer needs to be homed and calibrated following the process described in our previous work [49,50]. Briefly, the process involves three steps: (a) XYZ homing axes to position the printhead in the middle of the print bed; (b) Z-axis calibration to tune the distance between the nozzle and the printing bed; (c) pressure calibration (manually set through a lateral knob located on the bioprinter), to find the optimal pressure value that enables a proper flow of material.

#### 2.4.2. Definition of Printing Parameters and Shape Fidelity Assessment

Shape fidelity was assessed following the protocols suggested by Paxton and Schwab et al. [51,52] that we already implemented in our previous study [49]. Two tests were carried out based on two different geometries (i.e., serpentine and grid structures) to assess quantitatively the shape fidelity of SA-SF hydrogels. All printings were performed at room temperature (25 °C), and two conical nozzles of 0.41 and 0.25 mm (inner diameter) were used. Moreover, because of the time-variable behavior of the SF, each test was performed at two timepoints, i.e., at 7 and 14 days from SF solubilization. The materials were prepared the day before each timepoint.

A Petri dish was used as printing support. Three samples were printed for each structure (serpentine-like or grid) and formulation. The images were acquired with an iPhone 12 camera and processed using ImageJ software (National Institutes of Health, Bethesda, Maryland, USA) to extract the geometrical dimensions required by the protocol.

#### Serpentine-Like Structure

**Figure 1:**
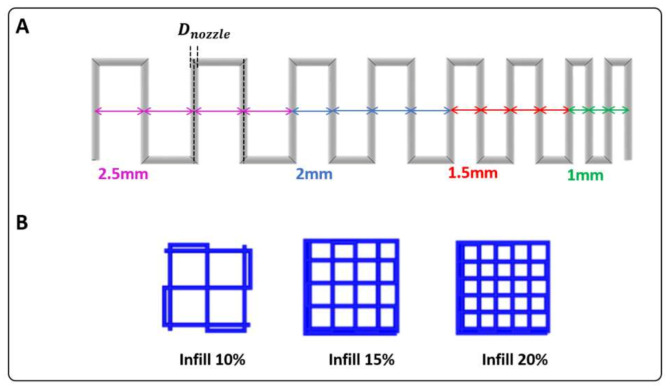
Geometries used for printability assessment: (**A**) serpentine structure; (**B**) grid structure with 10%, 15%, and 20% infill.

This test consisted of printing a single-layer and continuous strand of material following the path represented in Figure 1. Firstly, this test was used to select the optimal printing parameters (i.e., printing velocity and pressure). Then, having defined these values, we quantified the degree of printing accuracy (PA (%)) both for the strand thickness and for the minimum achievable distance between filaments, because adjacent filaments printed in the same layer can fuse. As a result, the degree of printing accuracy for (a) the filament width and (b) the minimal inter-filament distance was calculated as follows:

$$PA\;\left(\%\right)=\left(1-\frac{\left|\bar{x_{r}}-x_{i}\right|}{x_{i}}\right)\times 100$$

where $\bar{x_{r}}$ represents (a) the real width of the filament and (b) the real inter-filament distance measured with ImageJ at multiple locations of each printed serpentine structure, then averaged; $x_{i}$ represents (a) the ideal strand thickness, which corresponds to the relevant nozzle diameter ($D_{nozzle}$), and (b) the ideal inter-filament distance, which is equal to (see Figure 1):

$$d_{i}-D_{nozzle}$$

#### Grid Structure

Monolayered grid structures were printed with three different infills (10%, 15%, and 20%). Printability factor (Pr) was used as a parameter to describe the precision in printing porous structures, calculated as a function of the pore perimeter (P) and pore area (A) using the following Equation:

$$Pr=\frac{P^{2}}{16A}$$

Pr was calculated for each pore within the same grid structure and then averaged. High geometric accuracy results in a printability index of Pr = 1 (square transversal pore geometry), while Pr < 1 and Pr > 1 correspond to a rounder or irregularly shaped transversal geometry, respectively.

### 2.5. Hydrogel Mechanical Characterization

#### 2.5.1. Sample Preparation

Hydrogel formulations reported in Table 2 were mechanically characterized. The materials were prepared the day before each timepoint.

Specific molds were designed and manufactured with a commercial 3D FDM printer (Creality 3D technology, China) to prepare samples for compressive and tensile tests. For the compressive test, cylindrical geometry with a diameter of 8 mm and height of 4 mm was used, as reported in [53]; for the tensile test, according to ASTM standard F2900-11 [54], dog-bone geometry was used. Next, hydrogels were poured inside the molds and crosslinked for 1 h to ensure proper crosslinking in the whole sample. After that, samples were kept hydrated inside deionized water before testing. Finally, samples were measured (i.e., diameter/platen separation and cross-section/platen separation for compression and tensile tests, respectively) using digital calipers before being mechanically tested.

Different samples were prepared for each condition depending on the material’s availability, as described in Table 3. SF-SF hydrogels were evaluated 7 and 14 days after SF solubilization to investigate the impact of time on their behavior, and they were all crosslinked with 2% *w*/*v* CaCl_2_ + 20% *w*/*v* KCl + 5% *w*/*v* protamine solution. SA hydrogel (control) is not time-dependent. For the tensile test, two crosslinking solutions (2% *w*/*v* CaCl_2_ + 20% *w*/*v* KCl + 5% *w*/*v* protamine and 2% *w*/*v* CaCl_2_) were compared.

#### 2.5.2. Mechanical Tests

Tests were carried out at room temperature (25 °C) using an MTS Insight 30 electromechanical testing system (MTS System Corporation), equipped with a 250 N load cell, following the protocols reported below.

#### Compression

Mechanical properties of SA-SF hydrogel in different formulations were investigated by performing uniaxial compression tests [55]. A displacement-driven unconfined compression test was performed with preload and test speed set at 0.03 N and 0.1 mm/min [56], respectively. Compression tests were performed at a maximum compression of 2.5 mm, corresponding to 50% compression.

#### Tension

For tensile test experiments, the gripping pressure was set at 10 bar, and the sample surfaces were covered with cardboard to prevent sample sliding. All tensile tests were performed with a preload and test speed set at 0.01 N and 1 mm/min, respectively [54]. Tensile tests were performed at a maximum tension of 15 mm, corresponding to 50% tension, or up to sample break.

#### 2.5.3. Data Elaboration

Applied force and corresponding displacement were simultaneously recorded. Mechanical properties were computed as follows. Force-displacement data ranging from preload threshold to failure point were considered for analysis. In particular, the nominal stress (σ) was calculated as the ratio between the applied force and the unit area of the sample (σ = F/A), while the strain (ε) was calculated as the ratio between the dimensional variation and the initial height of the sample (ε = L − L_0_/L_0_). Finally, each stress–strain curve was fitted with a spline adopting a least-squares approach. Finally, the elastic modulus (E) was computed as the average of the spline derivative in the strain range between 0 and 0.1.

### 2.6. Release Study of Lyosecretome

#### 2.6.1. Scaffold Fabrication

Porous cylindrical hybrid scaffolds (d: 10.52 mm, h: 9.45 mm) of polycaprolactone (PCL) and lyosecretome-laden SA-SF hydrogel were designed and 3D co-printed according to our previous study [27]. Briefly, CELLINK INKREDIBLE+ was utilized to print in combination PCL pellets heated at 90 °C and lyosecretome-laden SA-SF hydrogel prepared by dissolving 20 mg of lyosecretome for each ml of the respective SA-SF hydrogels (Table 2). The following printing parameters were used: extrusion pressure 350 kPa; conical nozzle diameter 0.5 mm; printing speed 45 mm/min; and printing temperature 90 °C. The lyosecretome-laden SA-SF hydrogel was printed into one internal well of 6.52 mm diameter and 5.4 mm height using a conical nozzle with diameter of 0.41 mm. Printing pressure was set according to values selected during assessment of hydrogel printability and shape fidelity (Section 2.4.2). After printing, SA was crosslinked with 2% *w*/*v* CaCl_2_ and 5% *w*/*v* protamine, and 20% *w*/*v* KCl was applied to induce the conformational change of SF.

#### 2.6.2. Drug Release Studies

The release tests were conducted on scaffolds immersed in pH 7.2 phosphate-buffered saline (PBS, USP). The quantification of proteins was assessed using a BCA protein assay kit (from Thermo Fisher Scientific, Milan, Italy), while lipids were dosed using the Nile red method that was previously validated for this purpose [57]. The protein and lipid concentrations were extrapolated from a concentration vs absorbance plot obtained from standard protein solutions (bovine serum albumin) or standard lipid solutions (phosphatidylcholine) using a third-degree polynomial equation with *R*^2^ = 0.99. Both analyses were performed on aliquots of PBS withdrawn at fixed time intervals. After each removal, an equivalent amount of fresh PBS was added to maintain the sink conditions. The cumulative amount of released proteins or lipids was calculated as a percentage using Equation (1):

Cumulative amount of drug released (%) = C_i_/C_0_ × 100 (1)

where C_i_ is the amount of the proteins or lipids released at a definite time interval, and C_0_ is the loaded protein or lipid amount. All the experiments were conducted in triplicate.

#### 2.6.3. Drug Release Kinetic Study

As reported below, the in vitro drug release data were interpolated using different kinetic models.

Higuchi:

*F*(*t*) = *k* × *t*^0.5^ (2)

*F*(*t*) = 100 × (1 − C × exp ^(−*k* × *t*)^) (3)

where *F*(*t*) is the amount of drug dissolved at time *t* and *k* is the release constant. Equation (3) is Equation (2.12) from [58].

Peppas–Sahlin:

*F*(*t*) = *k_1_* × *t^m^* + *k*_2_ × *t*^(2 × *m*)^ (4)

where *F*(*t*) is the amount of drug dissolved at time *t*, *k*_1_ is the diffusion constant, *k*_2_ is the erosion constant, and *m* is the diffusional exponent, indicative of the drug-release mechanism.

Ritger–Peppas:

*F*(*t*) = *k* × *t^n^* (5)

where *F*(*t*) is the amount of drug dissolved at time *t*, *k* is the release constant, and *n* is the release exponent, indicative of the drug-release mechanism.

Zero-order:

*F*(*t*) = *k* × *t* (6)

where *F*(*t*) is the amount of drug released in time *t*, and *k* is the release constant.

Korsmeyer–Peppas:

*F*(*t*) = *k_KP_* × *t^n^* × Q_0_ (7)

where *F*(*t*) is the amount of drug released at time *t*; *k_KP_* is the release constant; *n* is the release exponent, indicative of the drug-release mechanism; and Q_0_ is the initial amount of drug.

### 2.7. Statistical Analysis

Raw data were processed using Statgraphics XVII (Statpoint Technologies, Inc., Warrenton, VA, USA). A generalized linear analysis of variance (ANOVA) model was used, followed by Fisher’s least significant difference (LSD) procedure to estimate the differences between the means. Comparisons among geometrical dimensions of the printability and shape fidelity tests were conducted using a one-way analysis of variance (ANOVA). Drug-release results were analyzed considering the batch as a fixed factor and the drug loading as the response variable. The statistical significance was set at *p* < 0.05.

## 3. Results and Discussion

The present work aimed to obtain an optimized SA-SF hydrogel for 3D bioprinting applications in the TE field. In terms of its ideal characteristics, this bioink should be easily printable with good mechanical properties. While the presence of fibroin in Silk I conformation is good for printability, because the low abundance of β-sheet network avoids clogging in the needles [44,45], its mechanical properties are not ideal; to this end, a Silk II conformation is indeed preferred to achieve high tensile strength and modulus [29]. Therefore, ideally, SF must (i) remain in Silk I conformation, i.e., in solution, during printing to prevent the nozzle from clogging, and then, after being printed, (ii) the conformational change from Silk I to Silk II must still be possible, to obtain a material with appropriate mechanical properties.

Regarding point (i), the degumming process (i.e., the separation of SF from sericin) was successfully modified. The standard degumming method (boiling the cocoons for 30 min in 0.02 M sodium carbonate) followed by fibroin dissolution in 9.3 M LiBr allowed us to obtain a regenerated SF that, despite being in Silk I conformation, was not printable, as the protein clotted the needle (data not shown). Therefore, the treatment with the alkaline solution was increased from the standard (30 min) to 1, 2, and 4 h. In agreement with previous reports [59], more hydrolysis of fibroin was observed: the SEC-UV analyses showed a decrease in the MWs of SF. Specifically, a broad peak was observed in each of the chromatographic profiles (Figure S1) of SF that had been degummed for 1, 2, and 4 h. The elution volumes calculated at the peak apex allowed estimation of the average MWs, reported in Table 4. Unlike the other samples, the 30 min degummed sample eluted mainly in the void volume, demonstrated to be outside of the top MW limit of the column (> 500 kDa). Peak area values showed acceptable precision for all the samples (RSD < 10%), even when repeatability diminished with reduced degumming time and thus diminished SF hydrolysis. This evidence may be related to precipitation and/or aggregation phenomena.

**Figure 2:**
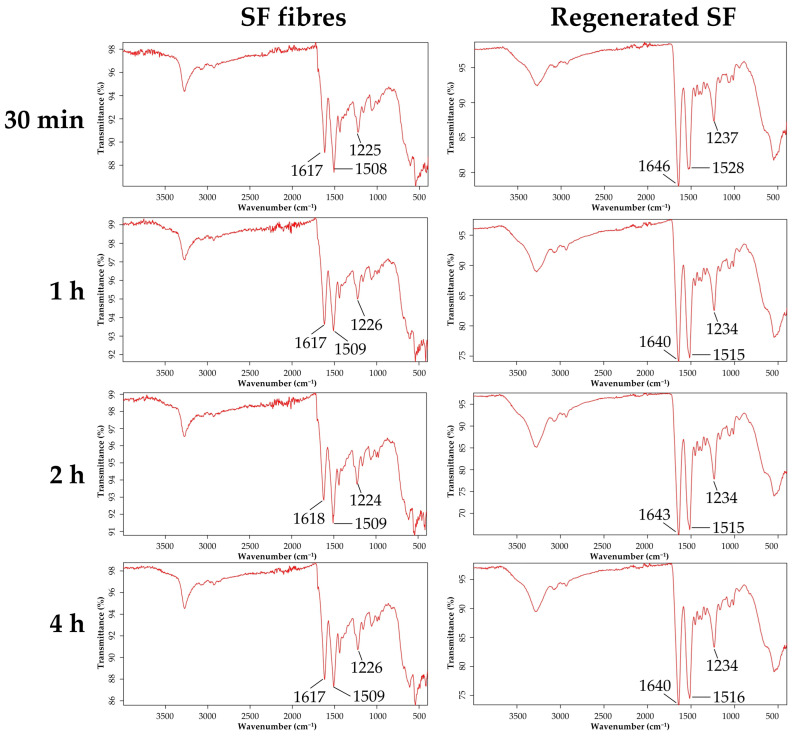
Infrared spectra of SF fibers degummed for 30 min, 1, 2, and 4 h, respectively, and regenerated SF treated with KCl 20% *w*/*v*.

Following this first step, regarding point (ii), it was assessed whether SF even with a low molecular weight could change its conformation from Silk I (in solution) to Silk II (insoluble). To evaluate this, the formation process of fibroin filaments in the silkworm was considered. Specifically, Chang and colleagues demonstrated that the pH gradient and cations in the anterior silk gland both play important roles in changing fibroin conformation: lower pH and Ca^2+^ levels and high K^+^ concentrations promote β-sheet formation [60]. Accordingly, fibroin samples were treated with KCl 20% *w*/*v* to induce conformational change from SilkI to Silk II. After being drying, the infrared spectra were recorded and compared with native SF fibers to assess the presence of the β-sheet network (Figure 2).

Amide I (1600–1700 cm^−1^) and amide II (1500–1600 cm^−1^) bands were used for the conformational analysis of the secondary structure. The absorption peaks in the amide I at around 1620 cm^−1^ are assigned to the β-sheet and at 1680 cm^−1^ to the random coil and/or helical conformation, while in the amide III bands, peaks at 1266 cm^−1^ and 1242 cm^−1^ are assigned to the β-sheet and random coil and/or helical conformation, respectively [61,62]. As observed in the spectra of the SF fibers, amide I and amide II bands were detected at about 1620 cm^−1^ (C = O stretching) and 1510 cm^−1^ (N-H bending), respectively, while amide III was present at about 1230 cm^−1^ (C-N and N-H functionalities). The amide I bands were still detectable for the regenerated SF treated with KCl but shifted towards 1640 cm^−1^, while the amide II and II bands remained unaltered. The shifting of amide I in the regenerated SF treated with KCl suggests a reduction of β-sheets and an increase in random coil conformations with respect to the native fibre. In this regard, it should be considered that SF chains have intramolecular or intermolecular interactions in which molecules are entangled. Therefore, it is likely that because of the reduced molecular weight following solubilization, the chain–chain interaction in the solution has been hindered, causing an increase of randomness in regenerated SF treated with KCl compared with the native fibers. However, no differences among the different degumming times were revealed, suggesting that all SFs were transited after treatment with KCl.

**Figure 3:**
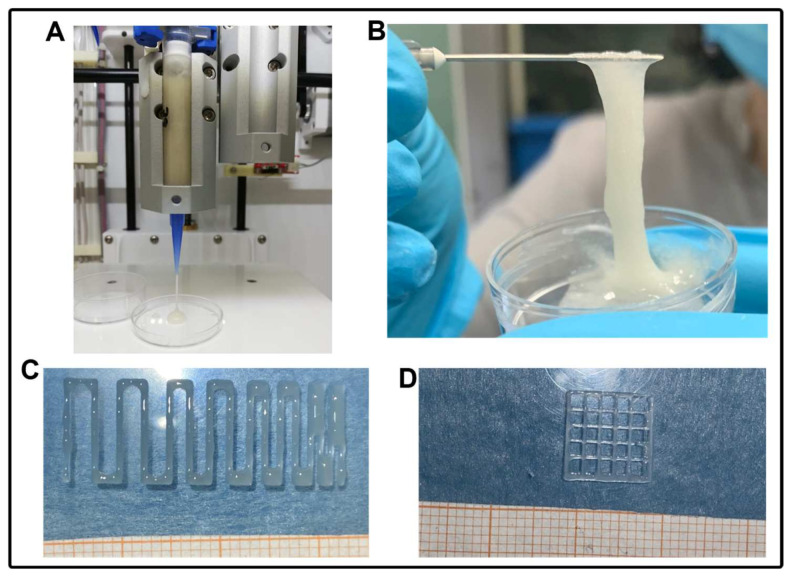
Printability assessment of the SA-SF hydrogels. (**A**) extrusion of the material using the 3D printer to set an adequate pressure value; (**B**) hydrogel appearance after printing; (**C**) and (**D**) examples of SA-SF hydrogel printed in serpentine structure and grid structure forms, respectively.

Then, a printing assessment was performed (Figure 3). To this end, the three different formulations of SA-SF hydrogels were evaluated in terms of shape fidelity at two different timepoints (7 and 14 days after SF production) to assess the time-dependent behavior of SF. Two different nozzle diameters (0.41 mm and 0.25 mm) were used during this characterization. Firstly, various combinations of printing speed and pressure were tested using a trial-and-error approach, as described in [51,52], with the final selection of the best pairs of values reported in Table 5.

It can be observed that 7 days after the preparation of SF, all three formulations were printable. The only difference was in SA-SF-1h, for which a lower pressure and a greater speed were selected; this is a typical feature of hydrogels with lower viscosity. However, 14 days after SF preparation, SA-SF-1h was not printable, probably due to a conformational change of the SF from Silk I to Silk II, leading the material to become stuck in the nozzle, thus making it hard to extrude [44,45]. Conversely, SA-SF-2h and SA-SF-4h were still printable with the same parameters determined at t1.

**Figure 4:**
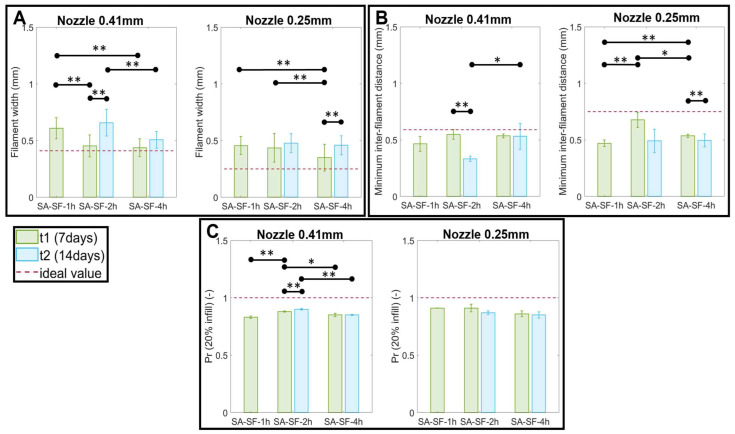
Results of shape fidelity assessment: (**A**) filament width; (**B**) minimum inter-filament distance (1 mm–D_nozzle_); (**C**) printability index. Data are reported as mean values ± SD. * *p* < 0.05, ** *p* < 0.01.

Then, once the printing parameters were defined, we quantitatively assessed the shape fidelity by measuring filament width, inter-filament distances, and the printability index (Pr) value. The following measures are shown in Figure 4. For inter-filament distance and the Pr, we report only the values related to the minimum resolution that we were able to reach, i.e., $1\;mm-D_{nozzle}$ and 20% infill, respectively.

Results show statistically significant differences in filament width, inter-filament distance, and Pr among the three formulations evaluated at the same timepoint with differences within the same formulation printed on different days after SF synthesis. Moreover, as expected, due to the pooling effect typical of viscous material such as hydrogel, it was impossible to satisfy the ideal values of diameter and inter-filament distance. All of the measured Pr values were slightly lower than the ideal value of 1, which describes good shape fidelity in the pores, while highlighting the viscous and potentially collapsible nature of the material.

Finally, as reported in Table 6, the printing performance of each SA-SF hydrogel was quantified. We can state that SA-SF-2h and SA-SF-4h, printed at the first timepoint with a 0.41 mm nozzle, showed the better results. However, at the second timepoint, there was a reduction in printing performance when the same speed and pressure values were maintained.

**Figure 5:**
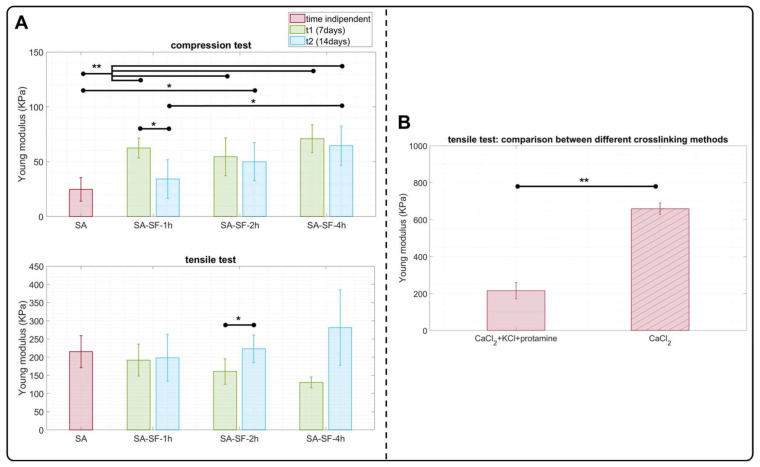
Mechanical characterization: (**A**) compressive and tensile results performed on SA hydrogel (control) and SA-SF hydrogels at 7 and 14 days from preparation of the silk-fibroin solution; (**B**) tensile analysis performed on SA hydrogels treated with CaCl_2_ + KCl + protamine solution and CaCl_2_ solution only. Data are reported as mean values ± SD. The number of samples for each condition is specified in Materials and Methods (Section 2.5.1). * *p* < 0.05, ** *p* < 0.01.

Mechanical evaluation was performed of SA-SF-based hydrogels characterized by different degumming times, and the results are shown in Figure 5. SA hydrogel was used as a control, with compressive and tensile elastic modulus values consistent with other scientific works [54,63]. Regarding the presence of SF in the hydrogel, ANOVA analysis showed that at t1 the degumming process did not affect the compression response for the three SA-SF formulations (*p* > 0.05), and their compressive moduli were three times higher (*p* < 0.01) than that of the hydrogel formed only with alginate, in accordance with previous findings [64]. At t2, SA-SF-2h and SA-SF-4h maintained their initial compressive modulus, while the compressive modulus of SA-SF-1h significantly decreased, reaching the Young’s modulus value of SA hydrogel. Conversely to the compression response, the presence of SF in SA hydrogel did not affect the tensile elastic modulus, and this was observed for each type of degummed SF at both timepoints (Figure 5B). Moreover, we performed a tensile test on SA hydrogels crosslinked with only 2% *w*/*v* CaCl_2_ and with 2% *w*/*v* CaCl_2_ + 5% *w*/*v* protamine + 20% *w*/*v* KCl to assess how the crosslinking method could influence tensile performance. As indicated in Figure 5C, the value significantly changed between the two crosslinking methods. Indeed, the CaCl_2_ solution formed a structure with a tensile modulus (~660 KPa) three times higher than the other method (~215 KPa). Therefore, it was hypothesized that during the crosslinking process the K^+^ ions of KCl compete with the Ca^2+^ ions in the formation of the alginate hydrogel, making it less compact overall. This observation suggested how to change the crosslinking method: at first with CaCl_2_, then with protamine, and only when completely gelified was the last treatment step performed with KCl. Analyzing the literature, we found that improvements in tensile performance for SF-based materials have mainly been obtained when fibroin is present in the form of fibers [65] or as physically crosslinked hydrogels [66]. However, no tensile mechanical characterization studies have been published for SF/SA blends chemically crosslinked with CaCl_2_, protamine, and KCl and few studies have characterized chemically crosslinked SF-based hydrogels with tensile testing. Indeed, as Yu Zhao et al. reported [67], chemical crosslinking leads to rapid gelification of the material without inducing in the SF network an immediate formation of β-sheet structures, which are responsible for conferring the structure’s high mechanical performance. In contrast, a physical crosslinking method stimulates the β-sheet’s nucleation, possibly reaching compressive and tensile modulus ranges of MPa.

**Figure 6:**
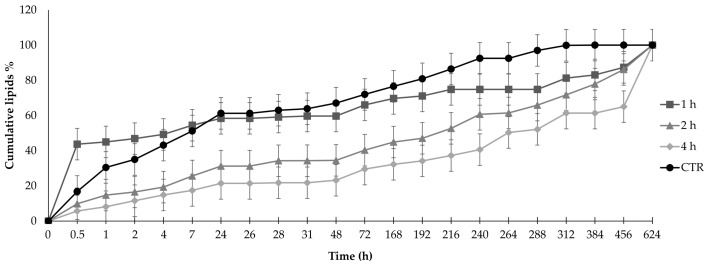
In vitro lipid release profiles from PCL scaffolds immersed in pH 7.2 phosphate-buffered saline (PBS) at room temperature. Comparison of PCL scaffolds without SF (CTR) and with SF degummed for different times (1, 2, or 4 h). Data are reported as mean values ± LSD, multifactor ANOVA, *n* = 3. The time on the x axis is not in scale, to better visualize the curves and the error bars. The overlap of two LSD intervals graphically indicates the absence of significant difference (*p* > 0.05).

Finally, we investigated how the presence of SF and its molecular weight modified the release of lyosecretome—a freeze-dried formulation of MSC secretome—from the SA-SF hydrogels. The results are reported in Figure 6 for only the lipidic component of the lyosecretome, i.e., the EVs, as it was impossible to discriminate if the protein released were of lyosecretome or fibroin. The time on the x axis is not in scale, to better visualize the curves and the error bars. The addition of SF significantly modified the release of lipids with respect to the baseline of SA-only hydrogel, which was considered a control, depending on the degumming time. Specifically, the release of EVs was faster than the control when 1 h degummed SF was used, while the use of SF degummed for 2 or 4 h significantly slowed the release of lipids (*p* < 0.001). In detail, after 2 h, SA-SF-1h hydrogel had a burst release of almost 50% vs 30% for the control and 16% and 11% for SA-SF-2h and SA-SF-4h, respectively.

The release data were further elaborated by the kinetic models employed, including Higuchi, Peppas–Sahlin, Ritger–Peppas, and zero-order, which were applied to investigate the release mechanism in greater depth (Table 7).

SA-SF hydrogels can control the release of EVs in lyosecretome by a combination of diffusion and erosion. According to this statement, the data fit the Ritger–Peppas and Korsmeyer–Peppas models with an R^2^ close to or greater than 0.9. The data also fit the Peppas–Shalin model, which considers a Fickian contribution (first term) and case-II relaxation (second term), i.e., diffusion and erosion, respectively [67,68]. Regarding the diffusion contribution (Ritger–Peppas and Korsmeyer–Peppas models), the diffusion speed decreases as the degumming time of the fibroin increases. For example, the K values were high when using 1 h degummed SF (29.56) and low (4.572, 1.982) when using 2 h and 4 h degummed SF, respectively. Unfortunately, as the results of the analyses were ambiguous, no further information can be drawn regarding the erosion mechanism. Furthermore, no interpretation of the release exponent (*n* in Equations (5) and (7) or *m* in Equation (4) is provided, as these exponents are valid only for drug-release systems with defined geometries (planar as thin films, cylindrical or spherical), which does not apply to the geometry of the scaffolds considered in this study. Based on observations of the effects of SF in the release profile, it is likely that its presence modifies the polymeric network and affects the interactions among polymer chains, depending on the molecular weight of SF. When the molecular weight of the protein is high, it offers a higher barrier to forming a compact polymeric network of alginate polymer chains, resulting in faster release. A key role in the diffusion process is played by the polymeric network and the steric hindrance it offers to the diffusion path of nanometric lipid assembly, including EVs. In general, an increase in the compactness of the polymeric network, consequent to a higher concentration of the polymer or higher interaction and crosslinking among polymer chains, slows diffusion. Conversely, when the molecular weight of SF is low, it does not hamper the formation of a solid and compact polymeric network of alginate polymer chains, and indeed the release is slower precisely due to the high steric hindrance the hydrogel offers. The slower release of SA-SF-2h and SA-SF-4h in comparison with the control may be due to the presence of SF in Silk II conformation reducing the erosion of the hydrogel, thus offering for longer a steric hindrance to lipid diffusion.

## 4. Conclusions

We optimized the preparation protocol for obtaining an SF-based solution by investigating three different degumming times to develop a 3D (bio)printable SA-SF-based hydrogel for TE applications. First, the extraction process of fibroin was optimized by increasing the degumming time in Na_2_CO_3_ 0.02 M (from 30 min to 1, 2, or 4 h) to reduce the fibroin’s molecular weight and thus achieve a printable protein solution that remained capable of the conformational change from Silk I (random coil) to Silk II (β-sheet). From a printing point of view, SA-SF hydrogels with a degumming time of 2 h and 4 h resulted in the best performance in term of shape fidelity. Furthermore, adding SF to the alginate hydrogel increased the compressive response, especially when degummed for 2 and 4h (e.g., at 7 days, Young’s modulus: 24.7 ± 10.7 KPa, 54.6 ± 17.3 KPa, and 70.9 ± 12.7 KPa for SA, SA-SF-2h, and SA-SF-4h hydrogels, respectively), probably caused by the formation of denser and condensed networks due to increased polymer content. However, it did not influence tensile performance (e.g., at 14 days, Young’s modulus: 215.5 ± 44.2 KPa, 198.6 ± 64.2 KPa, 223 ± 38 KPa, and 281.3 ± 103.8 KPa for SA, SA-SF-1h, SA-SF-2h, and SA-SF-4h hydrogels, respectively), for which a physical crosslinking method could be adopted for future development. Indeed, we found that the crosslinking method in the material strongly influenced the tensile response. For example, for SA hydrogel treated with CaCl_2_ + KCl + protamine solution and CaCl_2_ solution, Young’s modulus results were 215.5 ± 44.2 KPa and 659.4 ± 31.5 KPa, respectively. Finally, degumming of SF for 2 and 4 h dramatically slowed the EV release and modified the kinetics and mechanism of release with respect to the SA hydrogel baseline.

Overall, these results lay the foundation for further development of SA-SF bioinks with modulable mechanical and EV-release properties, and their use in scaffold 3D printing.

## Figures and Tables

**Table 1:** Overview of the used degumming methods.

Process	Solvent	Temperature	Time
Standard	Na_2_CO_3_ 0.02 M	100 °C	30 min
Short alkaline boiling			1 h
Medium alkaline boiling			2 h
Intensive alkaline boiling			4 h

**Table 2:** Overview of hydrogel formulations. SA: sodium alginate, SF: silk fibroin, CRTL: control.

Formulation	Formulation (*w*/*v*)	SF Degumming Time (h)
SA-SF-1 h	SA 10% + SF 5%	1
SA-SF-2 h		2
SA-SF-4 h		4
SA (CRTL)	SA 10%	/

**Table 3:** Number of samples for each mechanically tested condition.

Hydrogel	Condition	Compression	Tensile
SA-SF-1h	t1 (7 days)	4	4
	t2 (14 days)	6	5
SA-SF-2h	t1 (7 days)	6	4
	t2 (14 days)	6	5
SA-SF-4h	t1 (7 days)	6	2
	t2 (14 days)	6	4
SA (CTRL)	CaCl_2_ + KCl + protamine	6	5
	CaCl_2_	-	6

**Table 4:** Data from SEC-UV analysis of SF derived from different degumming times. Data are reported as mean values ± SD, *n* = 3.

Degumming Time (h)	Elution Volume ± SD (mL)	Estimated MW (Da)	Area ± SD	Area RSD (%)
0.5	2.724 ± 0.04	> 500 *	2011 ± 105	5.2
1	3.186 ± 0.05	174	2317 ± 75	3.3
2	3.581 ± 0.05	60	2235 ± 59	2.6
3	3.392 ± 0.05	24	2271 ± 18	0.8

* Outside of the MW range of the column (15–500 kDa).

**Table 5:** Optimized values of printing parameters (n.p. = non-printable).

Hydrogel	Printing Parameters	t1 (7 Days)		t2 (14 Days)	
		Nozzle 0.41 mm	Nozzle 0.25 mm	Nozzle 0.41 mm	Nozzle 0.25 mm
SA-SF-1h	Speed (mm/min)	1000	1000	n.p.	n.p.
	Pressure (kPa)	8	12	n.p.	n.p.
SA-SF-2h	Speed (mm/min)	600	600	600	600
	Pressure (kPa)	20	30	20	30
SA-SF-4h	Speed (mm/min)	600	600	600	600
	Pressure (kPa)	20	35	20	35

**Table 6:** Characterization of each formulation regarding the degree of PA% and Pr.

Hydrogel	Nozzle (mm)	t1 (7 Days)			t2 (14 Days)		
		PA (%) Filament Width	PA (%) Inter-Filament Distance	Pr	PA (%) Filament Width	PA (%) Inter-Filament Distance	Pr
SA-SF-1h	0.41	51.6	79	0.83	/	/	/
	0.25	17.7	63	0.9	/	/	/
SA-SF-2h	0.41	90	93	0.88	39.5	56.5	0.9
	0.25	25.7	90.3	0.91	9	65.4	0.87
SA-SF-4h	0.41	93.4	58	0.85	76.2	90	0.85
	0.25	60	91.4	0.86	16.3	66	0.85

**Table 7:** Results of release model fitting. Kinetic elaborations were performed on release data obtained from at least three independent experiments for each batch. ~ indicates that the analysis performed was “ambiguous”; therefore, the fit does not confirm the values of all the parameters, and 95% confidence bounds cannot be reported. These latter data were not considered in the interpretation of results.

Model	Equation	Formulation	SF Degumming Time (h)	Coefficients (95% Confidence Bounds)	Sum of Squares	R^2^	Degrees of Freedom
Higuchi	*F*(*t*) = *k* × *t*^0.5^	SF + alginate	1	k = 3.192 (2.525, 3.859)	7317	0.217	21
			2	k = 1.919 (1.776, 2.061)	334.3	0.8972	21
			4	k = 2.179 (1.996, 2.362)	550.9	0.9012	21
		Alginate	/	k = 1.394 (1.188, 1.600)	698.5	0.3735	21
Higuchi (eq 2.12 from [58])	*F*(*t*) = 100 × (1 − C × exp ^(−*k* ×^ *^t^*^)^)	SF + alginate	1	C = 0.6746 (0.6237, 0.7260) k = 0.00107 (0.0006436, 0.001540)	0.1290	0.6093	22
			2	C = 0.9037 (0.8825, 0.9250) k = 0.0009412 (0.0008108, 0.001075)	227.1	0.9301	22
			4	C = 0.9232 (0.8952, 0.9513) k = 0.001269 (0.001088, 0.001457)	389.7	0.9301	22
		Alginate	/	C = 0.887 (0.8605, 0.9137) k = 0.000429 (0.0002833, 0.0005791)	372.4	0.6659	22
Peppas– Sahlin	*F*(*t*) = *k_1_* × *t^m^* + *k*_2_ × *t*^(2 ×^ *^m^*^)^	SF + alginate	1	k_1_ = ~ 21.45 k_2_ = ~ 8.173 M = ~ 0.07298	115.1	0.9651	22
			2	k_1_ = ~ 4.311 k_2_ = ~ 0.6770 M = ~ 0.2624	137.7	0.9576	22
			4	k_1_ = ~ 2.304 k_2_ = ~ 0.1857 M = ~ 0.3911	461.5	0.9173	22
		Alginate	/	k_1_ = -0.1679 (-0.3920, -0.01953) k_2_ = 0.2406 (0.1669, 0.3539) m = 0.2023 (0.06861, 0.4205)	0.01727	0.9706	22
Ritger– Peppas	*F*(*t*) = *k* × *t^n^*	SF + alginate	1	k = 29.56 (27.43, 31.73) n = 0.09653 (0.08189, 0.1117)	121.1	0.9633	22
			2	k = 4.572 (3.189, 6.214) n = 0.3454 (0.2892, 0.4099)	163.9	0.9496	22
			4	k = 1.982 (0.7195, 4.194) n = 0.5166 (0.3834, 0.6913)	549.4	0.9015	22
		Alginate	/	k = 7.767 (6.967, 8.600) n = 0.1922 (0.1725, 0.2128)	26.66	0.9761	22
Zero-order	*F*(*t*) = *k* × *t*	SF + alginate	1	k = 0.1509 (0.09954, 0.2023)	15065	0.3564	22
			2	k = 0.09762 (0.08080, 0.1144)	1613	0.5038	22
			4	k = 0.1145 (0.1001, 0.1291)	1200	0.7849	22
		Alginate	/	k = 0.06667 (0.04768, 0.08566)	2057	0.2161	22
Korsmeyer– Peppas	*F*(*t*) = *k_KP_* × *t^n^* × *Q*_0_	SF + alginate	1	k_KP_ = 29.56 (27.43, 31.73) n = 0.09653 (0.08189, 0.1117)	121.1	0.9633	22
			2	k_KP_ = 4.572 (3.189, 6.214) n = 0.3454 (0.2892, 0.4099)	163.9	0.9496	22
			4	k_KP_ = 1.982 (0.7195, 4.194) n = 0.5166 (0.3834, 0.6913)	549.4	0.9015	22
		Alginate	/	k_KP_ = 7.767 (6.967, 8.600) n = 0.1922 (0.1725, 0.2128)	26.66	0.9761	22

## Data Availability

The data presented in this study are contained within the article.

## Supplementary Materials

The following supporting information can be downloaded at: https://www.mdpi.com/article/10.3390/pharmaceutics15020383/s1, Figure S1: SEC-UV profile of SF degummed for 30 min (blue trace), 1 h (red trace), 2 h (green trace), and 4 h (pink trace).
