# Supplementary material for: Silk Fibroin Bioink for 3D Printing in Tissue Regeneration: Controlled Release of MSC extracellular Vesicles
Source: Pharmaceutics. 2023 Jan 22;15(2):383. doi: 10.3390/pharmaceutics15020383 (PMC9959026; doi:10.3390/pharmaceutics15020383)
Supplement: Supplementary file 1 [file pharmaceutics-15-00383-s001.zip › pharmaceutics-2142442-supplementary.pdf]

Silk Fibroin Bioink for 3D Printing in Tissue Regeneration: Controlled Release of MSC-Extracellular Vesicles

Elia Bari, Giulia Maria Di Gravina, Franca Scocozza, Sara Perteghella, Benedetta Frongia, Sara Tengattini, Lorena Segale, Maria Luisa Torre and Michele Conti

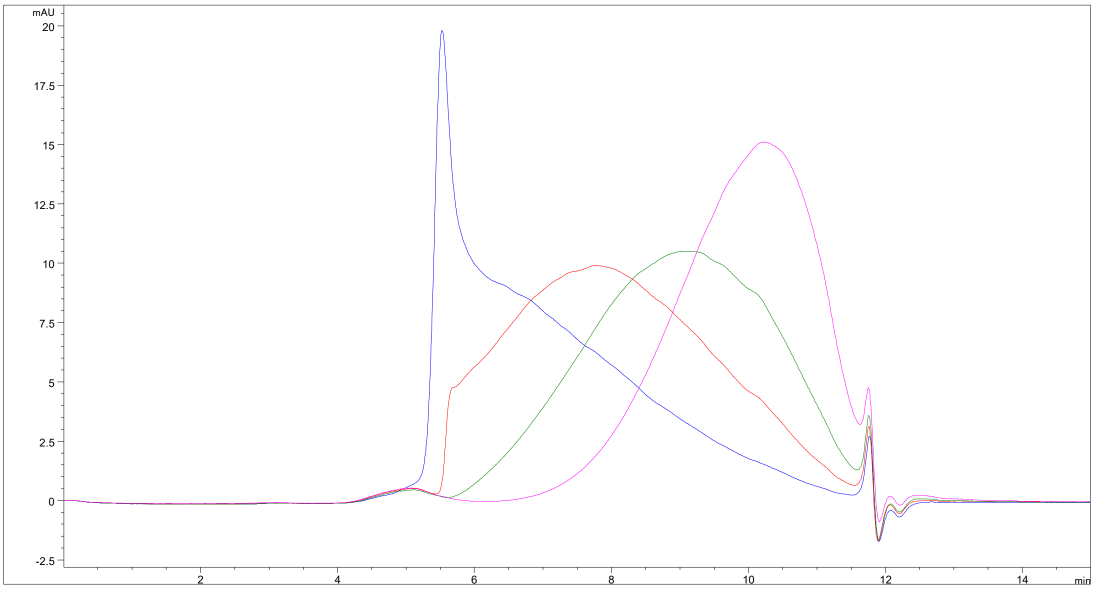

Figure S1. SEC-UV profile of SF degummed for 30 min (blue trace), 1 h (red trace), 2 h (green trace) and 4 h (pink trace).
